# *EWSR1::ATF1* fusions characterize a group of extra-abdominal epithelioid and round cell mesenchymal neoplasms, phenotypically overlapping with sclerosing epithelioid fibrosarcomas, and intra-abdominal *FET*::*CREB* fusion neoplasms

**DOI:** 10.1007/s00428-024-03879-5

**Published:** 2024-07-20

**Authors:** Bharat Rekhi, Josephine K. Dermawan, Karen J. Fritchie, Annette Zimpfer, Tareq M. Mohammad, Fatima S. Ali, Koushik Nandy, Youran Zou, Robert Stoehr, Abbas Agaimy

**Affiliations:** 1 Department of Pathology, Tata Memorial Hospital, Homi Bhabha National Institute (HBNI) University, Mumbai, Maharashtra India; 2 Department of Pathology and Laboratory Medicine Institute, Cleveland Clinic, Cleveland, OH USA; 3 Institute of Pathology, Rostock University Medical Center, Rostock, Germany; 4 Jaber Al-Ahmed Al-Sabah Hospital, Kuwait City, Kuwait; 5 Medica Superspecialty Hospital, Kolkata, West Bengal 700099 India; 6 Kaiser Permanente Oakland Medical Center, Oakland, USA; 7 Institute of Pathology, University Hospital Erlangen (UKER), Friedrich-Alexander University Erlangen-Nürnberg (FAU), Erlangen, Germany; 8 Comprehensive Cancer Center Erlangen-EMN (CCC ER-EMN), Erlangen, Germany

**Keywords:** Targeted next-generation sequencing, Ewing sarcoma, Precision medicine, Genetic landscape, Profiling, Mimics

## Abstract

With the increasing use of next generation sequencing in soft tissue pathology, particularly in neoplasms not fitting any World Health Organization (WHO) category, the spectrum of *EWSR1* fusion-associated soft tissue neoplasms has been expanding significantly. Although recurrent *EWSR1::ATF1* fusions were initially limited to a triad of mesenchymal neoplasms including clear cell sarcoma of soft tissue, angiomatoid fibrous histiocytoma and malignant gastrointestinal neuroectodermal tumor (MGNET), this family has been expanding. We herein describe 4 unclassified extra-abdominal soft tissue (*n* = 3) and bone (*n* = 1) neoplasms displaying epithelioid and round cell morphology and carrying an *EWSR1::ATF1* fusion. Affected were 3 males and 1 female aged 20–56 years. All primary tumors were extra-abdominal and deep-seated (chest wall, mediastinum, deltoid, and parapharyngeal soft tissue). Their size ranged 4.4–7.5 cm (median, 6.2). One patient presented with constitutional symptoms. Surgery with (2) or without (1) neo/adjuvant therapy was the treatment. At last follow-up (8–21 months), 2 patients developed progressive disease (1 recurrence; 1 distant metastasis). The immunophenotype of these tumors is potentially misleading with variable expression of EMA (2 of 3), pankeratin (2 of 4), synaptophysin (2 of 3), MUC4 (1 of 3), and ALK (1 of 3). All tumors were negative for S100 and SOX10. These observations point to the existence of heretofore under-recognized group of epithelioid and round cell neoplasms of soft tissue and bone, driven by *EWSR1::ATF1* fusions, but distinct from established *EWSR1::ATF1*-associated soft tissue entities. Their overall morphology and immunophenotype recapitulate that of the emerging *EWSR1/FUS::CREB* fusion associated intra-abdominal epithelioid/round cell neoplasms. Our cases point to a potentially aggressive clinical behavior. Recognizing this tumor type is mandatory to delineate any inherent biological and/or therapeutic distinctness from other, better-known sarcomas in the differential diagnosis including sclerosing epithelioid fibrosarcoma.

**Supplementary Information:**

The online version contains supplementary material available at 10.1007/s00428-024-03879-5.

## Introduction

The phenotypic and genotypic spectrum of sarcomas of the soft tissues and bones harboring *EWSR1* fusions has been rapidly expanding during the last two decades, as a consequence of widespread use of next generation sequencing (NGS) studies in routine practice and during research [1–4]. Recent studies have highlighted a variety of sarcomas harboring *EWSR1* gene rearrangements fused to non-*ETS* family of transcription factors [5, 6]. These tumors comprise a heterogeneous category of old and new or emerging entities including morphologically defined sarcomas/neoplasms with *EWSR1::CREB* fusions and unclassified sarcomas with non-*ETS EWSR1* fusions such as *EWSR1::PATZ1* [5, 6] and *EWSR1::NFATC2* fusions [6].

The phenotypic and genotypic landscape of mesenchymal neoplasms harboring *FET (EWSR1/FLI1)::CREB* fusions, which was initially described as the genetic landmark of clear cell sarcoma of soft tissue, has been growing and now includes angiomatoid fibrous histiocytoma (AFH) and analogous intrapulmonary and intracranial neoplasms [7], and malignant gastrointestinal neuroectodermal tumors (MGNET) [8] and their extra-abdominal counterparts [9]. Among the non-mesenchymal categories, *EWSR1::ATF1* fusions define the majority of hyalinizing clear cell carcinoma of head and neck [10] and have been reported also in a rare subset of mesotheliomas [11].

Recently, an emerging category of unclassified intra-abdominal neoplasms characterized by epithelioid morphology, frequent keratin expression, and recurrent *EWSR1/FUS::CREB(ATF1/CREM)* fusions have been reported as a potentially distinct entity by several groups [12–14]. The latter has remained ambiguously defined and has not been well delineated as an independent entity yet. We herein describe four unclassified extra-abdominal mesenchymal neoplasms showing epithelioid/round cell morphology and harboring *EWSR1::ATF1* fusions and review 6 additional cases carrying the same fusion, reported in three recent studies in a trial to delineate the major features of this putative tumor category.

## Material and methods

The 4 cases were identified in the consultation files of the authors. Unclassified morphology not fitting any defined WHO category and presence of NGS-verified *EWSR1::ATF1* fusion were the inclusion criteria. The tissue specimens were fixed in formalin and processed routinely for histopathology. Due to the consultation nature of the cases, immunohistochemistry (IHC) was performed in different laboratories and the stains applied varied from case to case, based on tissue availability and initial differential diagnostic considerations (details of the staining protocols and antibody sources are available upon request). The Medline literature was searched for soft tissue neoplasms carrying *EWSR1::ATF1* fusions and distinct from the above-mentioned well-defined WHO entities.

### Next-generation sequencing

For Cases 1 and 2, RNA was isolated from formalin-fixed paraffin embedded (FFPE) tissue sections and subjected to targeted RNA sequencing using the Illumina TruSight Oncology 500 RNA Panel (https://emea.illumina.com/products/by-type/clinical-research-products/trusight-oncology-500.html) as previously described [15]. Cases 3 and 4 were tested using an Anchored Multiplex PCR-based targeted RNA sequencing (Archer Dx) as previously described [16].

## Results

### Clinical features

**Fig. 1:**
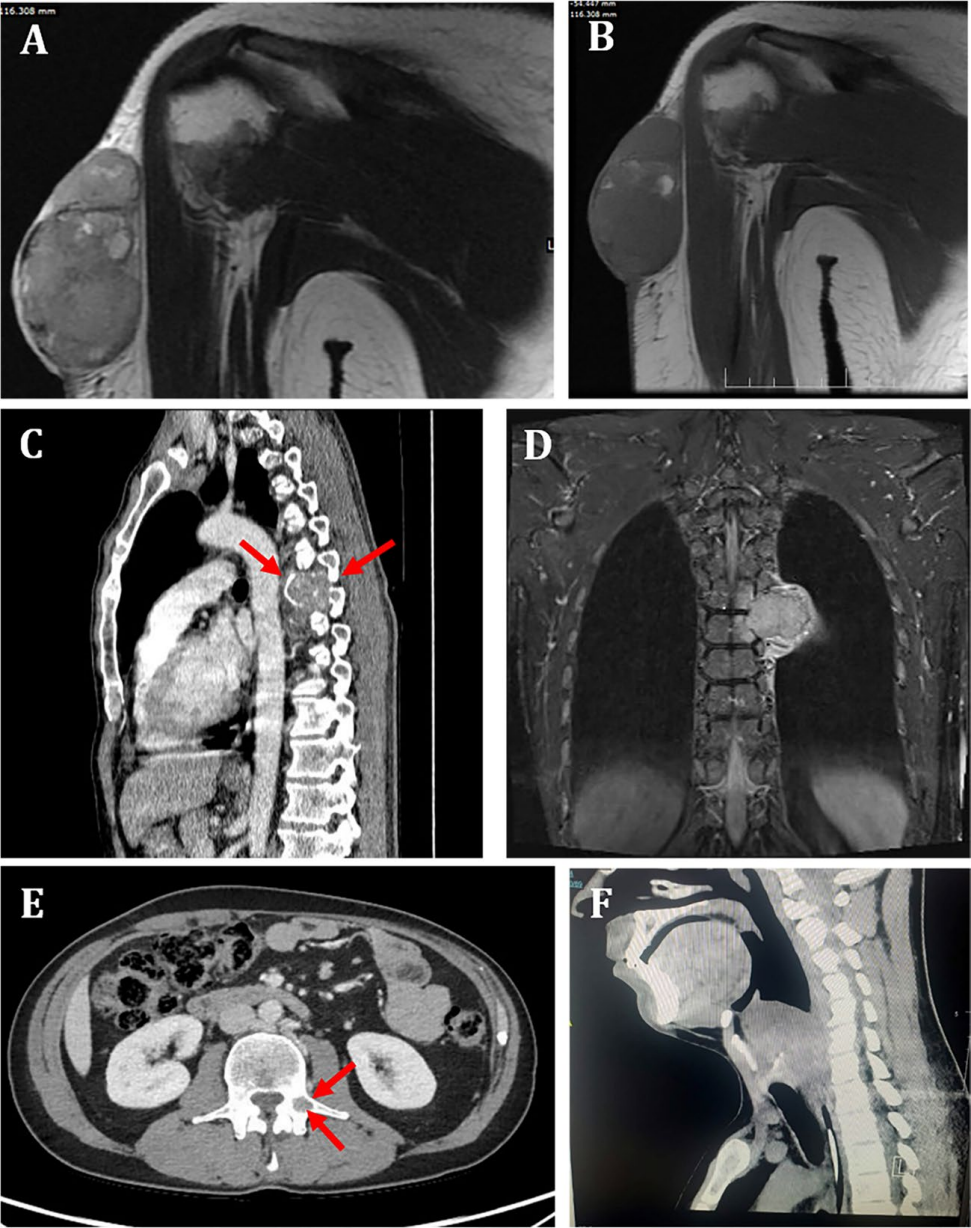
Representative imaging findings. **A** and **B** (Case 1): large, well-circumscribed, lobulated mass, isointense to muscles on T1 and heterogeneously hyperintense on T2-weighted images, measuring 7 cm cranio-caudally. **C** (CT) and **D (**MRI) in Case 2 revealed a large destructive mass (arrows) likely originating from the 7th rib and invading into the 6th and 7th thoracic vertebral bodies without intraspinal extension. **E** Progressive osteolytic metastases in same patient were detected in 2nd and 3rd lumbar vertebrae on follow up and verified by core needle biopsies (arrows). **F** CT of the head and neck in Case 4 revealed large neck soft tissue mass adjacent to the supraglottic larynx and the paralaryngeal tissue

Patients were three males and one female aged 20 to 56 years (median, 52) (Table 1). All tumors were extra-abdominal, located in the deltoid area, mediastinum, paralaryngeal/parapharyngeal soft tissue, and chest wall (Fig. 1). Involvement of adjacent bone at the chest wall was noted in Case 2. Tumor sizes ranged from 4.4 to 7.5 cm (mean, 6.2). A localized mass was the main clinical symptom in three patients (although clinical and serological details were not available in these three case). One patient (Case 3) with detailed clinical findings presented with constitutional symptoms including fever of unknown origin, elevated ESR, CRP, and ferritin with mild normocytic anemia and thrombocytopenia. This patient presented with a mediastinal tumor that was locally invasive into the left ventricular wall and likely into the left atrium. Surgery was the treatment in three patients (Cases 1, 2, and 4), of which one (Case 1) received neoadjuvant chemotherapy and the other (Case 2) received adjuvant radiotherapy. One patient (Case 3) received systemic chemotherapy only. The fourth patient was a recent case with no details on further treatment modalities. Two patients with follow-up developed disease progression; one (Case 1) developed local recurrence at 8 months, and the other (Case 2) was diagnosed with histologically verified vertebral body metastasis 21 months from primary diagnosis.

**Table 1:** Clinical features of *EWSR1::ATF1* fusion unclassified epithelioid/round cell neoplasms

No	Age/sex	Site/size	Size (cm)	Symptoms	Original diagnosis	Treatment	Outcome
1	32/F	Right deltoid	7.5	Mass	Neuroblastoma, then revised to unclassified round cell sarcoma of neuroectodermal type (not Ewing sarcoma)	Chemotherapy, then surgery Surgery + CRT for recurrence	Local recurrence (8 mo)
2	48/M	Chest wall	7	Mass	Unclassified (CREM-like) epithelioid/round cell sarcoma with *EWSR1::ATF1* fusion	Surgery + 50 Gy + boost radiotherapy with 10 Gy	Vertebral metastasis L3 (21 mo)
3	56/M	Mediastinum invading the left ventricular wall and possibly extending into the left atrial lumen	4.4	Fever of unknown origin, elevated ESR, CRP, and ferritin with mild normocytic anemia and thrombocytopenia	Unclassified round cell neoplasm	Adriamycin, ifosfamide, and mesna chemotherapy	NA
4	20/M	Paralaryngeal (supraglottic) soft tissue	5.9	Mass	Unclassified (CREM-like) epithelioid/round cell sarcoma with *EWSR1::ATF1* fusion	Surgery	Very recent case

*CRT* chemoradiotherapy, *mo* months, *NA* not available

### Pathological findings

**Fig. 2:**
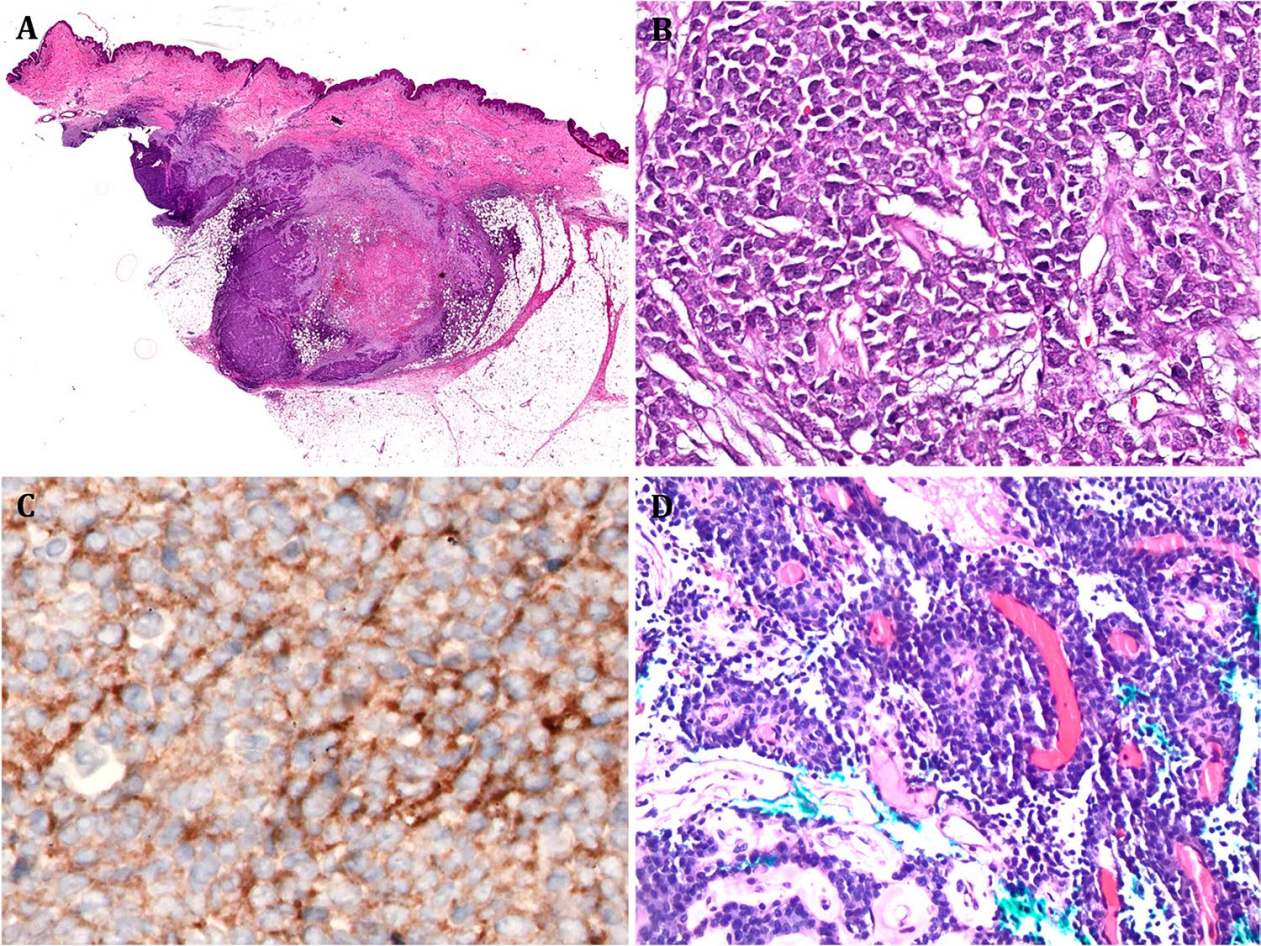
Representative images of **A–C** Case 1 and **D** Case 3. **A** Overview image of the recurrent tumor in Case 1 showing a subcutaneous infiltrating nodular tumor with extensive areas of central necrosis. **B** Monomorphic basophilic cells with discohesive diffuse arrangement are seen at high power (same case). **C** The same tumor expressed diffusely synaptophysin. **D** Prominent perivascular pseudopapillary growth resulting from cell discohesion is seen in Case 3

**Fig. 3:**
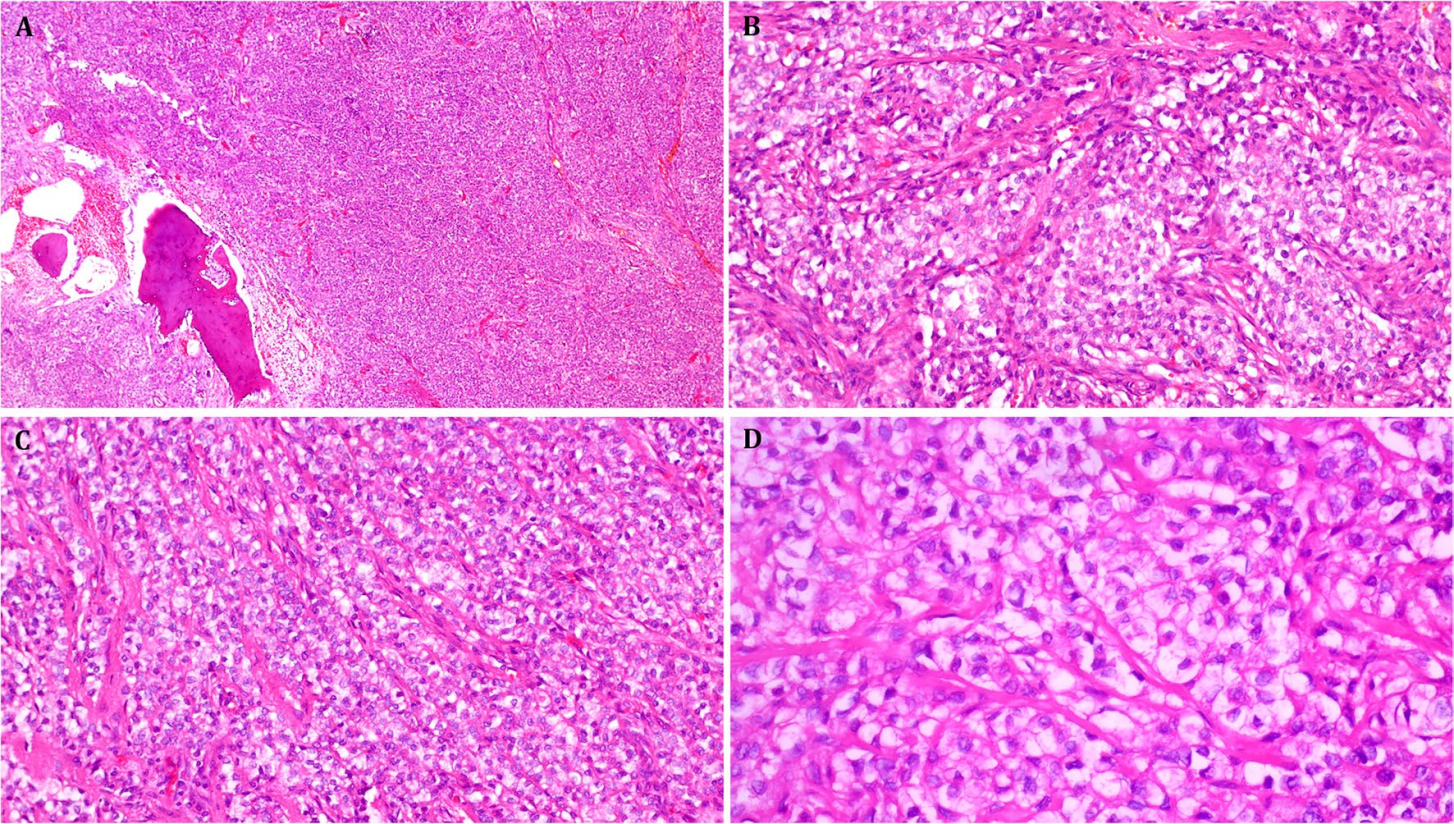
Representative images of Case 2. **A** Case 2 revealed monomorphic cellular neoplasm invading the adjacent bone of the chest wall. **B** The neoplastic cells were disposed into organoid nests interrupted by fibrous septae. **C** Other areas showed compressed thin trabeculae with abortive single cell file-like arrangement of cells. **D** Organoid packaging of cells and prominent cytoplasmic clearing is seen at higher magnification

**Fig. 4:**
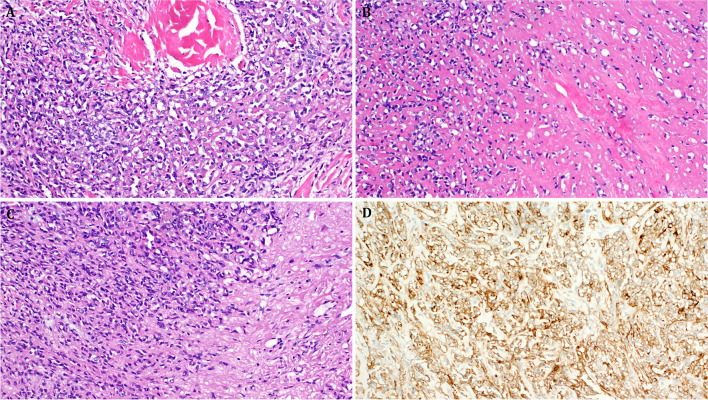
Representative images of Case 4. Case 4 showed the most striking similarity to sclerosing epithelioid fibrosarcoma with **A** fine-reticular fibrosis entrapping single or aggregates of few cells and **B** confluent amorphic hyaline fibrosclerosis. **C** Focal spindling of cells with transition to sclerosis. **D** Strong and diffuse expression of EMA

All primary tumors were deep-seated or involved skeletal muscle. The local recurrence in Case 1 was subcutaneous (Fig. 2). All cases were sent for second opinion with putative diagnoses of neuroblastoma, undifferentiated/unclassified round cell tumors, and remained unclassified even after expert second opinion due to the lack of a fitting/defined WHO category. Microscopic examination revealed poorly circumscribed tumors composed of monotonous medium-sized round cells. The cells possessed isomorphic nuclei with heterogeneous chromatin and lacked prominent nucleoli. The cytoplasm varied from scant and amphophilic/basophilic (Cases 1 and 3; Fig. 2) to moderate and clear to pale-eosinophilic (Cases 2 and 4; Figs. 3 and 4). The tumor cells were arranged into diffuse sheets and solid aggregates with frequent organoid architecture/nesting within scant variably myxocollagenous to highly fibrous stroma, showing some similarities to neuroendocrine neoplasms and sclerosing epithelioid fibrosarcoma (SEF). Focal discohesive arrangement with perivascular pseudopapillary structures was seen in Case 3 (Fig. 2D). Mild mononuclear chronic inflammation was noted in Case 2, including focal aggregates at the tumor periphery, but lymphoid cuffs, syncytial cell growth, aneurysmal changes and myxoid features were absent in all cases. Cases 2 and 4 showed more resemblance to sclerosing epithelioid fibrosarcoma and had focal spindling of cells (Figs. 3 and 4). The mitotic activity ranged from 2 to 12 mitoses/10 HPFs. Foci of necrosis were observed in Case 2.

### Immunohistochemical findings

**Fig. 5:**
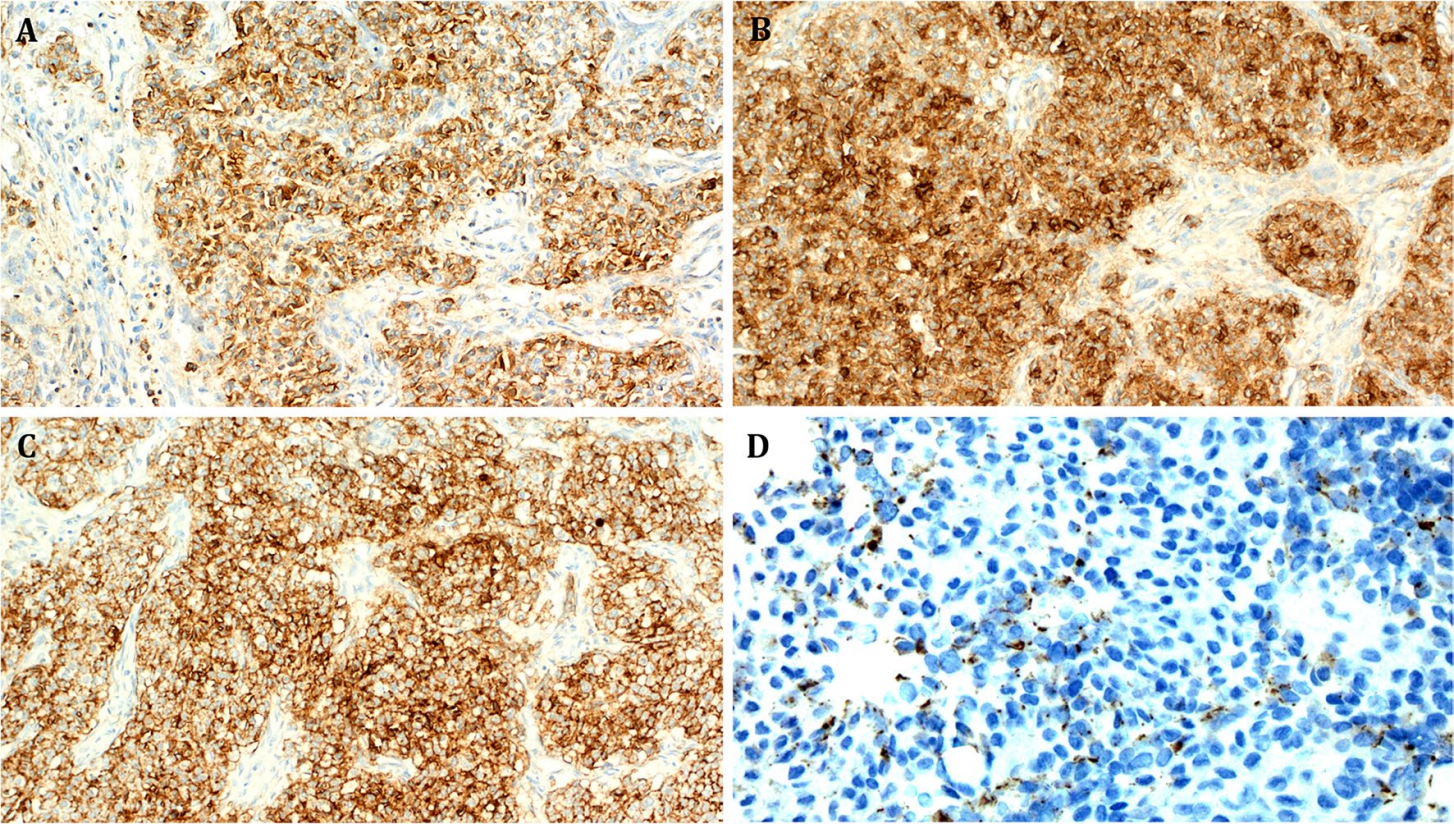
Representative images of immunohistochemical findings in **A–C** Case 2 and **D** Case 3. **A** Heterogeneous weak to moderate, mostly cytoplasmic expression of CD99. **B**: strong and diffuse expression of EMA. **C** Strong diffuse reactivity for MUC4. **D** Paranuclear dot-like keratin reactivity

The immunohistochemical findings were generally nonspecific. The neoplastic cells expressed at least one neuroendocrine marker in 2 of 3 tested cases: 2 of 3 were positive with synaptophysin, 2 of 2 were positive with CD56, and one tested case was positive for INSM1. Variable expression of epithelial markers was another feature with pankeratin/AE1/AE3 expressed in 2 of 4 cases and EMA in 2 of 3 cases. Focal ALK expression combined with expression of MUC4 was noted in one of 3 cases. All tumors were negative for S100 (0/4), SOX10 (0/4), and desmin (0/3). All other markers listed in Table 2 were negative. Representative images of the immunohistochemical results are depicted in Figs. 2C, 4D, and 5A–D.

**Table 2:** immunohistochemical and molecular findings in *EWSR1::ATF1* fusion unclassified epithelioid/round cell neoplasms

No	Mitoses/10 hpfs	IHC +	IHC-	Breakpoint/exon EWSR1	Breakpoint/exon ATF1
1	12/10 hpfs	Synaptophysin, INSM1, CD56	NKX2.2, chromogranin, MUC4, ALK, CK20, AE1/AE3, and desmin, WT1, CD99	*EWSR1* (NM_005243.4) exon 8	*ATF1* (NM_005171.5) exon 4
2	2/10 hpfs	MUC4, EMA, CD56, synaptophysin, CD99, focal ALK	S100, Chromogranin, TFE3, Stat6, SATB2, NUT, CD34, ERG, AE1/AE3, Pan-Melanoma, SOX-10, Desmin, CD10	*EWSR1* (NM_005243.4) exon 7	*ATF1* (NM_005171.5) exon 5
3	6/10 hpfs	Pankeratin (focal dot-like)	SMA, CK20, TTF1, p40, PAX8, desmin, myogenin, SALL4, beta-catenin, S100, SOX10, HMB45, MelanA, CD10, CD34, synaptophysin, chromogranin, calretinin, STAT6, EMA, WT1	*EWSR1* (NM_005243.4) exon 8	*ATF1* (NM_005171.5) exon 4
4	12/10 hpfs	EMA, patchy AE1/3	ALK, MUC4, SOX10, S100, p63, ERG, CD31, MyoD1	*EWSR1* (NM_005243.4) exon 11	*ATF1* (NM_005171.5) exon 2

### Molecular findings

Case 1 was tested initially by a FISH probe targeting the *EWSR1* rearrangement, which revealed red-green split signals in 96% of the tumor cell nuclei, indicating *EWSR1* gene rearrangement (Supplementary Fig. S1). However, reverse transcriptase polymerase chain reaction (RT-PCR), failed to show any positive fusion transcripts for *EWSR1::FLI1*, *EWSR1::ERG*, and *EWSR1::WT1* fusions (all negative). This justified subsequent targeted RNA fusion panel (TruSight panel, Illumina) testing which revealed the *EWSR1::ATF1*-fusion. All other cases were subjected to targeted RNA fusion panel (TruSight panel, Illumina or Archer AMP-based RNA sequencing) testing during primary reporting as they did not fit into any defined sarcoma category. All four tumors revealed an *EWSR1::ATF1* fusion. The fusion breakpoints mapped to *EWSR1* exon 8 (2 cases), exon 7 (one case), or exon 11 (one case), which was fused to *ATF1* exon 4 (2 cases), exon 2 (one case), or exon 5 (one case) (Table 2).

## Discussion

Fusions between members of the *FET* gene family (*EWSR1* and *FUS*) and genes encoding for the CREB-transcription factor family (*ATF*, *CREB1*, and *CREM*) have been reported in a variety of clinically and pathologically distinct neoplasms of mesenchymal [1–4, 12–14], epithelial [10] and mesothelial [11] origin. *EWSR1::ATF1*-driven mesenchymal entities encompass phenotypically diverse mesenchymal neoplasms with melanocytic (clear cell sarcoma of tendon and aponeuroses (CCS)) [17], neuroectodermal-like (malignant gastrointestinal neuroectodermal tumor (MGNET) [8, 9], and undefined/unknown (angiomatoid fibrous histiocytoma (AFH) [18], primary pulmonary myxoid sarcoma [19], and primary intracranial myxoid sarcoma [20])) histogenesis/phenotype. Moreover, the *EWSR1::ATF1* fusion is characteristic of hyalinizing clear cell carcinoma of the head and neck (HCCC; defined by distinctive morphology and epithelial/squamous cell immunophenotype) [10] and has been reported in a rare subset of conventional mesothelioma with predominance of epithelioid morphology and early age of onset [11].

Recently, *EWSR1::CREM* fusions have been reported in a novel tumor type characterized by epithelioid/round cell morphology, predominance of intra-abdominal location, lack of neuroectodermal/melanocytic immunophenotype, frequent expression of epithelial markers [12–14] and lack of defining features of the well-known *EWSR1/FUS::CREB* fusion entities [12–14]. Notably, 6 of these recently reported intra-abdominal neoplasms harbored an *EWSR1::ATF1* fusion as alternate to the most frequent *EWSR1/FUS::CREM* fusion, but were otherwise indistinguishable from the *CREM*-fusion-positive category [12–14].

We herein describe a unique cohort of soft tissue neoplasms characterized by epithelioid/round cell morphology, unusual immunophenotype, and recurrent *EWSR1::ATF1* fusions, all occurring in extra-abdominal deep soft tissue or bone sites. All cases were sent for second opinion with putative diagnoses including neuroblastoma and undifferentiated/unclassified round cell tumors and remained unclassified even after expert second opinion due to the lack of a fitting/defined WHO category, highlighting the potential difficulty in classifying them. Despite the shared *EWSR1::ATF1* genotype, the morphology and immunoprofile of this cohort are quite distinct from the known *FET::CREB* fusion entities.

Including our study, 10 unclassified mesenchymal neoplasms with epithelioid/round cell morphology and an *EWSR1::ATF1* fusion have been reported (Tables 3 and 4) [12–14]. Affected patients were 6 males and 4 females aged 20 to 62 years (median, 40). All previously reported tumors were intra-abdominal, originating from the peritoneum and visceral abdominal organs (Table 3). Our current 4 cases represent the first extra-abdominal documentation of this tumor type. Their size range was 4.4–8.2 cm (median, 5.3). Four of 8 (50%) patients with clinical details presented with constitutional symptoms. Surgery with (1) or without (7) adjuvant therapy was the treatment, except in two patients who received systemic chemotherapy. At last follow-up (8–25 months), 4 of 8 patients (50%) developed progressive disease (3 recurrences and one distant metastasis), one patient was alive with persistent disease on chemotherapy at 14 months, while three were disease-free at 9, 10 and 16 months. One patient died of disease at 25 months. The progressive cases were similarly distributed to extra- (2) and intra-abdominal (3) sites.

**Table 3:** Clinicopathological features of previously reported and current *EWSR1::ATF1* fusion unclassified epithelioid/round cell neoplasms

No	Ref	Age/sex	Site	Size (cm)	Symptoms	Treatment	Outcome
1	Argani et al., Case 9 [12]	62/M	Peripancreatic	5.3	NA	Surgery	NED (9 mo)
2	Argani et al., Case 13 [12]	36/F	Rectovaginal pouch	NA	NA	Surgery	Peritoneal recurrence (unspecified period)
3	Shibayama et al. [13]	54/M	Intraabdominal	6.4	Fever, anemia, weight loss, nicht sweat, elevated CRP	Chemotherapy	AWD (14 mo)
4	Shibayama et al. [13]	34/M	Ileum	8.2	Anemia, increased serum CRP	Surgery (Chemotherapy for recurrence)	Rec (16 mo), DOD (25 mo)
5	Trecourt et al., Case 1 [14]	41/F	Left uterine adnexa	5.2	Fever, anemia, weight loss, inflammatory syndrome with increased serum CRP, shock	Salpingo-oophorectomy	NED (16 mo)
6	Trecourt et al., Case 2 [14]	39/F	Ovary	NA	Mass	TAHBSO	NED (10 mo)
7	Current Case 1	32/F	Right deltoid	7.5	Mass	Surgery (chemotherapy + radiation therapy for recurrence)	Local recurrence (8 mo)
8	Current Case 2	48/M	Chest wall	7	Mass	Surgery + 50 Gy + boost radiotherapy with 10 Gy	Vertebral metastasis L3 (21 mo)
9	Current Case 3	56/M	Mediastinum invading left ventricle	4.4	Fever, elevated ESR, CRP, and ferritin, mild normocytic anemia and thrombocytopenia	Adriamycin, ifosfamide, and mesna chemotherapy	On follow-up
10	Current Case 4	20/M	Supraglottic	NA	Mass	Surgery	Very recent case

*AWD* alive with disease, *mo* months, *NA* not available, *NED* no evidence of disease

**Table 4:** Immunohistochemical and molecular findings in previously reported and current *EWSR1::ATF1* fusion unclassified epithelioid/round cell neoplasms

No	Ref	CK	EMA	Synaptophysin	Chromogranin	MUC4	ALK	Desmin	S100	SOX10	EWSR1 breakpoint	ATF1 breakpoint
1	Argani et al., Case 9 [12]	NA	pos	neg	neg	neg	pos	NA	neg	NA	Exon 7	Exon 5
2	Argani et al., Case 13 [12]	AE1/AE3 & cam5.2 pos	pos	neg	neg	neg	neg	NA	neg	NA	Exon 14	Exon 5
3	Shibayama et al. [13]	AE1/AE pos	NA	neg	NA	pos (focal)	NA	neg	NA	NA	NA	NA
4	Shibayama et al. [13]	AE1/AE pos	NA	pos (focal)	neg	pos (focal)	pos (focal)	neg	neg	NA	NA	NA
5	Trecourt et al., Case 1 [14]	AE1/AE3& cam5.2 neg	pos	neg	neg	pos (focal)	neg	pos	neg	neg	Exon 6	Exon 4
6	Trecourt et al., Case 2 [14]	AE1/AE3& cam5.2 pos	pos	pos	pos	pos	pos	pos (focal)	neg	neg	Exon 6	Exon 4
7	Current Case 1	AE1/3 neg	NA	pos	neg	neg	neg	neg	neg	neg	Exon 8	Exon 4
8	Current Case 2	AE1/3 neg	pos	pos	neg	pos	pos (focal)	neg	neg	neg	Exon 7	Exon 5
9	Current Case 3	Pankeratin pos	neg	neg	neg	NA	NA	neg	neg	neg	Exon 8	Exon 4
10	Current Case 4	AE1/AE3 pos	pos	NA	NA	neg	neg	NA	neg	neg	Exon 11	Exon 2

*NA* not available, *neg* negative, *pos* positive

The immunophenotype of these tumors is unusual and misleading. These tumors have in common frequent co-expression of epithelial and neuroendocrine markers associated with variable reactivity with ALK and MUC4. Altogether, either low molecular weight keratins, EMA or both were expressed in 9 of the 10 reported tumors (90%). Four of 8 cases (50%) expressed synaptophysin and one of them coexpressed chromogranin. MUC4 and ALK were expressed in 5 of 9 (55%) and 4 of 8 (50%) cases, respectively. All cases tested negative for S100 (0/9) and SOX10 (0/9). The differential diagnosis in these cases is heterogeneous and challenging from case to case and includes diverse entities. Among these differential diagnoses, five merit special consideration.

First, AFH is well known for its varied morphology and may show areas with round cell or epithelioid morphology. However, AFH is a disease of the young with its peak in the first two decades of life, in contrast to the median age of 40 years in the entity we are discussing. Moreover, AFH, if strictly diagnosed by pertinent criteria, only rarely behave aggressively. Notably, all of our cases and the 6 recently reported intra-abdominal cases lacked all of the key features of AFH and none was myxoid. Desmin was only focally positive in 2 of 7 tumors, in contrast to AFH, which typically stains for desmin in > 50% of cases, while being consistently negative for cytokeratin. Admittedly, the morphological overlap among these *FET::CREB* fusion entities would not be a surprise as shared genotypes frequently have their subtle fingerprints in otherwise morphologically, histogenetically, and biologically distinct entities. The site distribution and the biological behavior of our cases are not compatible with AFH. More importantly, AFH was not a consideration in any of the cases, although all were seen by expert soft tissue pathologists who judged them as unclassified into any of the known WHO categories by morphology and immunophenotyping.

Second, based on the predominance of round cell morphology in some cases (as in our Case 1), these tumors need to be distinguished from other round cell sarcomas including Ewing and Ewing-like sarcomas harboring *EWSR1::Non-ETS* (*EWSR1::PATZ1* and *EWSR1::NFATC2*) fusions. In this context, overdependence on *EWSR1* FISH testing can be misleading. These tumors do not show the homogeneous membranous CD99 expression, and they lack the stereotypical cytology of classical Ewing sarcomas. Moreover, their histology and immunophenotype differ from *EWSR1::Non-ETS-*rearranged sarcomas harboring *EWSR1::PATZ1* and *EWSR1::NFATC2* fusions (for more details on these very rare entities the reader is referred to ref. [6]).

Third, the morphology of these tumors and the frequent coexpression of keratins/EMA and neuroendocrine markers represent a major source of confusion with neuroendocrine neoplasms. Notably, chromogranin as the most reliable neuroendocrine marker in excluding mimics of neuroendocrine neoplasms [21] has been rarely reported in these tumors [22, 23]. However, characteristic morphology (insular, trabecular, pseudoacinar, gyriform, and corded architecture and salt-and-pepper chromatin pattern) is more reliable in recognizing genuine neuroendocrine neoplasms. Review of the depicted histological images in a recently reported paraganglioma with *EWSR1::CREM* fusion showed overlapping features with our cases [23].

Fourth, distinguishing these mesenchymal neoplasms from HCCC at head and neck sites (as in our Case 4) is another challenge. Their variable clear cell morphology, EMA/keratin expression, and the variable sclerosis may be confused with HCCC by the unexperienced. However, the cytological pattern and architecture of these mesenchymal neoplasms, the stromal hyalinization pattern, and lack of squamous immunomarkers (CK5, p63/40) are sufficient to distinguish the two entities. Indeed, the confusion might only emerge after staining for keratins. These cases highlight how epithelial and mesenchymal entities originating at similar anatomic sites may share variable morphological/immunophenotypic and genotypic features, still being unrelated and significantly distinct biologically.

Fifth, distinguishing *FET::CREB* fusion neoplasms reported herein from other well-defined *EWSR1* fusion entities in the morphological spectrum of SEF-like neoplasms requires more than MUC4 immunohistochemistry and *EWSR1* FISH testing. Most SEFs are driven by *EWSR1::CREB3L1* (rarely *FUS::CREB3L1)* fusions, and they express MUC4 [24, 25]. Accordingly, MUC4 expression in tumors with SEF-compatible morphology is still considered sufficient adjunct to confirm SEF [25]. A small subset of MUC4-negative SEF-like sarcomas harbors recurrent *YAP1::KMT2A* fusions [26, 27]. Our study adds a third genetic tumor category with a variable SEF-like morphology with/or without MUC4 expression driven by *FET::CREB* fusions [28, 29]. While no specific treatment has been established for any of these recently delineated genotypic SEF-like sarcoma subcategories, their distinction seems justified to address potentially different pathobiology and treatment responsiveness. Emerging novel therapeutic strategies targeting *EWSR1::ATF1/CREM* fusions represent yet another argument for separating them [30, 31].

Finally, a distinctive aggressive testicular neoplasm carrying a *EWSR1::ATF1* fusion has been reported recently under the descriptive name “inflammatory and nested testicular sex cord tumor” [32, 33]. The authors claimed a sex cord origin, based on frequent expression of SF1 and α-inhibin. However, their illustrations suggest at least in part variable histological similarity to intra-abdominal *FET::CREB* fusion mesenchymal neoplasms and our current cases [12–14]. Moreover, they showed frequent expression of EMA, keratin and CD30, and some expressed chromogranin [32, 33]. While the testicular tumors were not tested for ALK and MUC4, recent reports of histologically similar ovarian tumors confirmed MUC4 expression [14, 34]. Whether these gonadal neoplasms are part of the *EWSR1/FUS::ATF1/CREM* fusion-driven tumor spectrum with subtle organ-specific phenotypic differences remains controversial. One of the authors has observed peritoneal *FUS::CREM* fusion neoplasms containing areas indistinguishable from the “inflammatory and nested testicular sex cord tumors” (Agaimy, unpublished data).

In summary, we described four extra-abdominal round cell/epithelioid mesenchymal neoplasms sharing *EWSR1::ATF1* fusions, frequent expression of epithelial and neuroendocrine markers, occasional aberrant expression of ALK and MUC4, but not fitting with any currently defined mesenchymal neoplasm. Promiscuity of *EWSR1::ATF1* fusions as well as the polyphenotypic phenotype of this tumor type may lead to diagnostic confusion with both mesenchymal and non-mesenchymal neoplasms. Even though follow-up data is limited, and the low number of cases precludes any conclusive results, these tumors are capable of aggressive behavior including distant metastasis, suggesting they behave as sarcomas. While the exact site-independent nosology of these entities is beyond the scope of the current study, the exact classification of these highly overlapping but differently named neoplasms from different organs/sites needs clarification as more cases from their different reported sites are recognized. This should then enable better characterization of their shared as well as distinct features.

## Supplementary Information

Below is the link to the electronic supplementary material.

Supplementary file1 (PNG 3750 KB)
