# Supplementary figures and images for: EWSR1::ATF1 fusions characterize a group of extra-abdominal epithelioid and round cell mesenchymal neoplasms, phenotypically overlapping with sclerosing epithelioid fibrosarcomas, and intra-abdominal FET::CREB fusion neoplasms
Source: Virchows Arch. 2024 Jul 20;485(6):995–1005. doi: 10.1007/s00428-024-03879-5 (PMC11666693; doi:10.1007/s00428-024-03879-5)

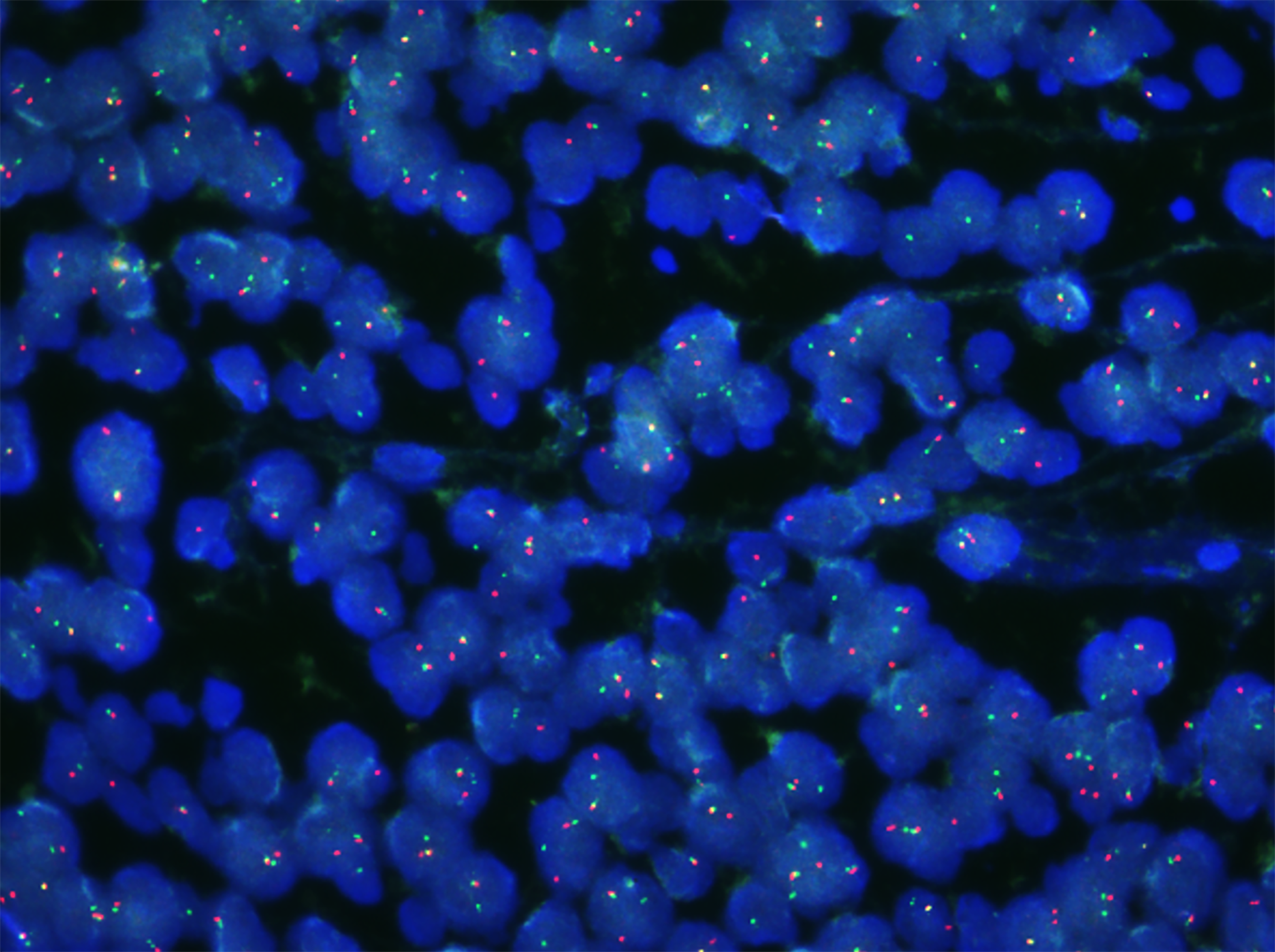

Supplement: Supplementary file 1 — Supplementary file1 (PNG 3750 KB) [file 428_2024_3879_MOESM1_ESM.png]
